# Significant Differences in RNA Structure Destabilization by HIV-1 Gag∆p6 and NCp7 Proteins

**DOI:** 10.3390/v12050484

**Published:** 2020-04-25

**Authors:** Micah J. McCauley, Ioulia Rouzina, Jasmine Li, Megan E. Núñez, Mark C. Williams

**Affiliations:** 1 Department of Physics, Northeastern University, Boston, MA 02115, USA; 2 Department of Chemistry and Biochemistry, The Ohio State University, Center for Retroviral Research, and Center for RNA Biology, Columbus, OH 43210, USA; 3 Department of Chemistry and Program in Biochemistry, Wellesley College, Wellesley, MA 02481, USA

**Keywords:** HIV-1, TAR hairpin, Gag, nucleocapsid, optical tweezers, energy landscape, transition state, mfold

## Abstract

Retroviral nucleocapsid (NC) proteins are nucleic acid chaperones that play distinct roles in the viral life cycle. During reverse transcription, HIV-1 NC facilitates the rearrangement of nucleic acid secondary structures, allowing the transactivation response (TAR) RNA hairpin to be transiently destabilized and annealed to a complementary RNA hairpin. In contrast, during viral assembly, NC, as a domain of the group-specific antigen (Gag) polyprotein, binds the genomic RNA and facilitates packaging into new virions. It is not clear how the same protein, alone or as part of Gag, performs such different RNA binding functions in the viral life cycle. By combining single-molecule optical tweezers measurements with a quantitative mfold-based model, we characterize the equilibrium stability and unfolding barrier for TAR RNA. Comparing measured results with a model of discrete protein binding allows us to localize affected binding sites, in addition to quantifying hairpin stability. We find that, while both NCp7 and Gag∆p6 destabilize the TAR hairpin, Gag∆p6 binding is localized to two sites in the stem, while NCp7 targets sites near the top loop. Unlike Gag∆p6, NCp7 destabilizes this loop, shifting the location of the reaction barrier toward the folded state and increasing the natural rate of hairpin opening by ~10^4^. Thus, our results explain why Gag cleavage and NC release is an essential prerequisite for reverse transcription within the virion.

## 1. Introduction

**Figure 1:**
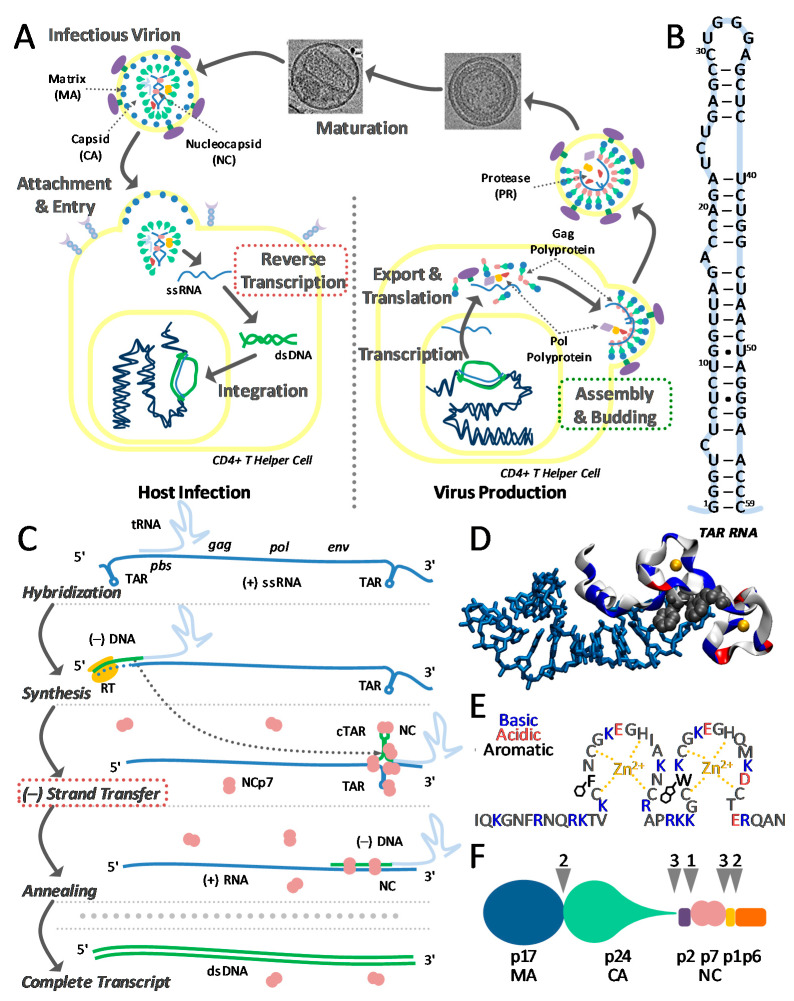
Critical steps in the viral life cycle. (**A**) In a standard picture of the infection, the virion attaches to a host cell and releases the viral RNA, which must undergo reverse transcription before integration. An infected cell will transcribe viral polyproteins, including the group-specific antigen (Gag). Polymerizing upon the host cell wall, Gag packages two RNA strands into the nascent virus. Reverse transcription may not occur until the nucleocapsid domain NCp7 is cleaved from Gag. (**B**) The folded structure of the 59-base transactivation response (TAR) RNA hairpin is highly irregular, with several bulges and mismatches. (**C**) Nucleic acid chaperone NCp7 facilitates minus strand transfer, a rate-limiting step in reverse transcription, where viral RNA (blue line) is converted into DNA (green line). (**D**) The sequence of NCp7, highlighting two zinc finger domains, responsible for nucleic acid binding and destabilization, and a basic tail (blue residues) responsible for chaperone activity. (**E**) NMR structure of the nucleocapsid (NC) bound to SL3, where two aromatic residues (black) stack with exposed bases (PDB code 1a1t) [17]. (**F**) Progressive cleavage (arrows) of Gag polyprotein by viral protease-free NCp7 as well as the matrix (MA) and capsid (CA) domains [18,19].

DNA and RNA hairpins participate in many critical rate-limiting biochemical processes [1,2,3,4,5]. These sequences are often chaperoned by ligands that facilitate the formation of a diverse set of secondary and tertiary structures to suit their roles [2,3,4,5,6,7,8]. The transactivation response (TAR) sequence in the HIV-1 virion forms a hairpin, which is involved in several key steps in the virion life cycle (Figure 1A), including transcription and reverse transcription [9,10]. This 59-base hairpin has an unusually irregular structure (Figure 1B) where the base pairing is interrupted by several bulges and mismatches. Despite the ‘patterned’ structure of the folded hairpin, it is stable enough to facilitate the faithful transcription of the remaining genome [9]. Once the virion infects a new host, however, the hairpin must open to facilitate the key step of minus strand transfer (Figure 1C) [11,12,13,14,15,16]. TAR unfolding in this rate-limiting step must proceed without the assistance of any ATP-driven motor.

The nucleocapsid protein NCp7 consists of 55 amino acids, forming two functional subdomains (Figure 1D). A basic tail serves as an effective aggregator of DNA and RNA. The other subdomain coordinates two zinc ions, often referred to as zinc ‘fingers’, which contribute to nucleic acid binding and destabilization. Each ‘finger’ also contains an aromatic residue capable of stacking with exposed residues (this is shown for the SL3 hairpin in HIV-1 in Figure 1E) [17]. Together, these subdomains combine to form a highly effective nucleic acid chaperone, which enables the rearrangement of nucleic acid structures into an optimal form [20,21,22]. In a previous study, we showed that NCp7 effectively destabilizes TAR and facilitates hairpin opening by a factor of 10^4^ [23]. However, NCp7 does not significantly affect the final annealed DNA–RNA hybrid [7,11].

Within an infected host cell, NCp7 is expressed as a subdomain of the group-specific antigen (Gag) protein. This polyprotein comprises four major domains: a matrix protein, a capsid protein, the nucleocapsid protein NCp7, and the viral budding p6 domain. (Figure 1F) [18]. Gag polymerizes on the host cell plasma membrane to form an immature virion, while packaging the viral RNA (Figure 1A). Between 1500–3000 Gag molecules are required to assemble a single virion [24,25,26]. Once the virion buds from the host, viral protease, contained as a subdomain within the Gag-Pol polyprotein (which constitutes about 5% of all Gag molecules), cleaves itself and subsequently NCp7 from the polyprotein [18]. Full cleavage of the polyproteins marks the conversion of the virus from immature to a mature, infectious virion (Figure 1F). Both NCp7 and Gag are effective at annealing ${\mathrm{tRNA}}_{3}^{\mathrm{Lys}}$ to the primer-binding site on the HIV-1 genome [27], which is the first step in reverse transcription that requires nucleic acid chaperone activity [10]. However, only processed nucleocapsid (NC) facilitates minus-strand transfer [28,29]—the next step in reverse transcription that requires effective nucleic acid chaperone activity [30,31]. Why is NCp7 a more effective chaperone than its parent protein, Gag, for minus strand transfer, despite NC being the primary RNA-interacting domain for both proteins?

In this work, we use single molecule force unfolding data to measure the free energy landscape of TAR hairpin unfolding. We then quantify changes to this landscape in the presence of the Gag∆p6 polyprotein. Energy landscapes are also modeled using data from mfold for the TAR sequence. Quantitative comparisons between the model and experiment allow us to deduce specific Gag binding sites along the TAR hairpin that alter the free energy of hairpin unzipping. We find that, while binding does destabilize the folded hairpin, it does not affect the lower stem, so that the transition barrier to unfolding is nearly unchanged and the natural rate of hairpin unfolding is increased by only ~10×. This result is in sharp contrast to the effect of NCp7: once cleaved from Gag, NCp7 binds to the TAR hairpin, where it not only destabilizes the folded state, but also uniquely shifts the location of the barrier to initiate unfolding. That shift means only a short stem needs to be unfolded, which leads to a decisive increase of ~10^4^× in the rate of unfolding [23]. Gag cleavage by the protease is thus a critical step that enables reverse transcription within the virion.

## 2. Materials and Methods

### 2.1. Hairpin Construct Synthesis

The creation of end labeled RNA hairpin/DNA handle hybrids has been outlined before [23,32]. Essentially, a 5′-biotinylated 3400 base pair DNA handle and a 3′-dixogygenin 3100 base pair DNA handle were each created from custom primers in PCR-amplified plasmid pBR322. The 5′-biotinylated and 3′-dixogygenin handles were digested with EcoRI and BSPEI, respectively, and then each was purified on a 0.8% agarose gel. A PCR-amplified 103-base synthetic DNA template was prepared to include the 59-base TAR sequence, the T7 promoter site and two flanking sequences. After transcription by T7 RNA polymerase, the resulting RNA was purified on a denaturing 10% PAGE gel. The RNA hairpin was ligated to short DNA oligos, using T4 RNA ligase 1 on the 5′ side and T4 RNA ligase 2 on the 3′ side (this required three ribonucleotides on the 3′ side of the oligo). Each ligation was followed by purification on a denaturing 10% PAGE gel. This construct was ligated to each handle with T4 DNA ligase, with a 0.8% agarose gel after each ligation. Finally, these TAR hairpin constructs were then incubated with a solution of streptavidin-coated beads (3.1 microns in diameter, Spherotech, Lake Forest, IL, USA), to achieve an average ratio of six constructs per bead.

### 2.2. Gag Polyprotein Preparation

Gag polyproteins studied here specifically lack myristic acid at the N-terminus and the p6 domain at the C-terminus and are also commonly referred to as Gag∆p6-Myr (though, throughout this work, they will be termed Gag∆p6) [33,34,35]. Gag∆p6 was prepared as previously described [33,34,35].

### 2.3. Single Molecule Optical Tweezers

**Figure 2:**
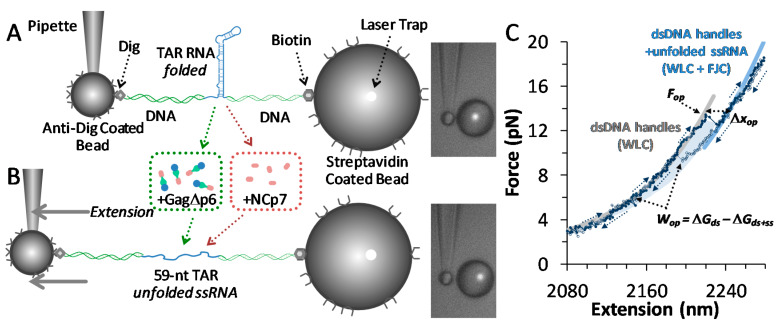
Single molecule assay observes hairpin opening and folding. (**A**) Single molecule construct of a TAR RNA hairpin (blue) flanked by long, labeled dsDNA handles (green), suspended between functionalized beads. The anti-dig-coated bead (2.1 μm diameter) was fixed onto a glass micropipette, while the streptavidin-coated bead (3.1 and 5.4 μm diameter beads were used) was held in a dual-beam optical trap. (**B**) Stepwise increases in extension raise the tension until a critical force is reached and the hairpin is completely disrupted, leaving ssRNA. (**C**) Three cycles of increasing force extension (along the arrows, filled circles) and force release (open circles decreasing along the arrows) reveal this length change (∆*x_op_*), the force (*F_op_*) and the work done by the instrument in hairpin unfolding (*W_op_*). Models of polymer elasticity are shown and are described in Appendix A. Experimental buffers included 10 mM HEPES (pH 7.5) and 100 mM Na^+^, unless noted. Results were compared to experiments performed in saturating conditions of Gag∆p6 and NCp7.

Several papers have detailed the optical tweezers setup and have specified the utility of this design for quantifying hairpin unfolding [23,36,37,38,39,40,41]. Here, a custom-built flow cell was placed at the center of a dual 830 nm beam (Lumics, Berlin, Germany), counter propagating optical trap. Anti-dig-coated beads (2.1 microns in diameter, Spherotech) flowed into the cell, were caught in the trap and pulled onto a pre-pulled micropipette tip (0.5-micron diameter, WPI). Beads pre-incubated with the TAR hairpin (~6 hairpins for each bead) were diluted (~fM), then introduced into and caught in the trap. The experimental buffer included 10 mM HEPES, pH 7.5 and 100 mM Na^+^ (no Mg^2+^ was used), while the flow cell temperature was 22 °C These experimental conditions are identical to experiments done on NCp7. Single tethers were isolated between the two beads and tested by force extension in the schematic shown in Figure 2A. Cycles of force extension and release were observed with a step resolution of 2 nm, at a corresponding loading rate of 10 pN/s. Figure 2B shows discrete hairpin opening events, which were characterized by opening and closing forces (*F_op_* and *F_cl_*) and an opening length (∆*x_op_*). The work performed by the instrument during unfolding was also determined as *W_op_* = ∆*G_ds_* − ∆*G_ds+ss_* (the shaded region in Figure 2B), which corrects for the elastic stretching contribution of both the hairpin and the DNA handles. Bead + TAR hairpins were also exposed to 100 nM and 10 nM solutions of Gag (in the same experimental buffer, which was also the same buffer used in NCp7 experiments). Hairpin opening statistics were not measurably different in these concentrations, while the DNA handles were somewhat less affected in the lower concentration. This is consistent with previous Gag–nucleic acid binding experiments that suggest that TAR RNA is saturated with the protein [35]. Because the incubated bead + TAR RNA hairpin solution is very dilute (~fM) compared to Gag∆p6 (10 nM), it is not the protein:RNA ratio, but rather the protein–RNA binding affinity determined by the solution’s ionic strength that determines the complex saturation that is required for protein function.

### 2.4. Hairpin Energies from Mfold

Theoretical landscapes were deduced from the base pair and stacking data set available at the mfold server [42,43]. Specifically, the RNA folding server (at http://unafold.rna.albany.edu), version 3.6, is given the 59-base TAR sequence (the UNAfold server also returns the same values under the appropriate conditions). Though the input conditions were fixed at 37 °C and 1 M Na^+^, we have previously shown that this is effectively equivalent to the experimental conditions of 22 °C and 100 mM Na^+^ in this study [23]. For the case of TAR RNA, only one hairpin structure corresponded to the minimum folded structure energy, which incorporated the full ∆*x_f_* = 59 bases (no unfolded flanking tails were left) with a total energy of ∆*G_f_* = 32.9 kcal/mol, or 55.5 k_B_T (this structure may be seen in Figure 1B). Furthermore, mfold provided a sequence of energies corresponding to each paired base (*N_i_*), including pairing and stacking contributions determined from an array of calorimetry studies; ∆*G_i_*(*N_i_*, *F*=0). These individual elements are summed to give the total hairpin energy, Σ*_i_*∆*G_i_*(*N_i_*, *F*=0) = *G_N_*(*N*, *F*=0) = ∆*G_f_*, which we used to construct the energy landscapes shown below.

## 3. Results

Parameters extracted from optical tweezer experiments are analyzed to create a landscape of TAR hairpin unfolding. Simulated landscapes from mfold are directly compared to the data to confirm the validity of this technique. The landscapes obtained in the presence of Gag∆p6 are compared to models of destabilization to determine the location and specificity of protein binding. Finally, Gag∆p6 binding is directly compared to the ability of NCp7 to dramatically enhance the opening rate of the TAR hairpin, which was shown in our previous work [23].

### 3.1. TAR Hairpin Length Is Unaffected by Gag∆p6

**Figure 3:**
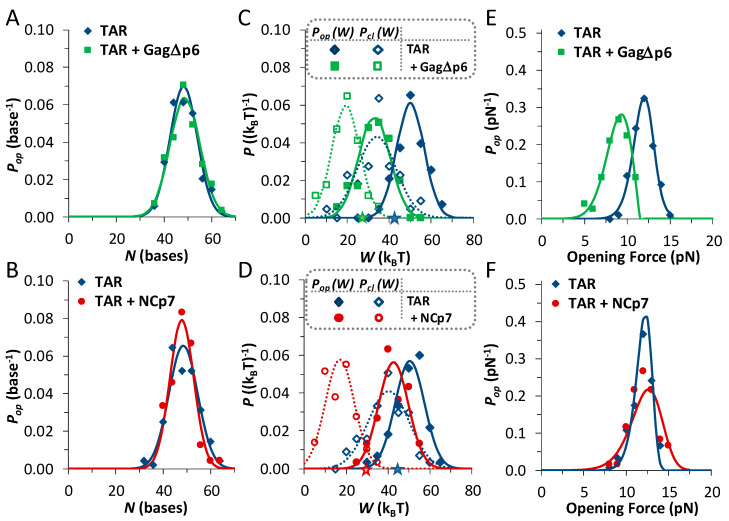
Force-extension/release data quantifies the energy and the barrier of unfolding. (**A**) The measured unfolding length (∆*x_op_*) for the TAR hairpin (blue, *n* = 86 events) and for TAR in the presence of 10 nM Gag∆p6 (green, *n* = 72). (**B**) The length for TAR alone (blue, *n* = 120) and in the presence of 50 nM NCp7 (red, *n* = 60), with no measurable change for either protein from the native hairpin. (**C**,**D**) The probability distributions of the work of unfolding/folding (solid/open symbols) cross at the energy of the hairpin unfolding (∆*G_o_*, marked with stars placed along the axis). Both (**C**) Gag∆p6 and (**D**) NCp7 destabilize the TAR hairpin. (**E**,**F**) Distributions of the opening forces fit to the transition state model described in the text (solid lines) determining the distance to the opening barrier ($\Delta x_{op}^{†}$) and the barrier height ($\Delta G_{op}^{†}$). Data in panels (**B**,**D**,**F**) were measured under the same conditions (as part of a larger data set) as in a previous work [23]. Quantified results are summarized in Table 1.

Force extension curves show a clear ‘rip’ due to the hairpin unfolding. The length of the unfolded hairpin is measured between the onset of the opening event (*F_op_*, found on Figure 2C), and the same force on the unfolded construct. Subtracting the extension difference between the same forces on these curves automatically eliminates changes in the elasticity of the handles and hairpin at different forces from the result. This result for the hairpin opening length (∆*x_op_*) is still a force-dependent length, and correcting for polymer elasticity yields a force-independent length change in the bases, plotted in Figure 3A. Appendix A details these models, which includes the Worm-Like Chain (WLC) used to describe dsDNA, while the Freely Jointed Chain (FJC) characterizes ssRNA. For the TAR hairpin, the unfolding length is measured to be 48.2 ± 0.6 bases. This measured length is shorter than the 59-base TAR sequence, and the discrepancy is due to the unusual amount of hairpin fraying, as explained below. Furthermore, this length did not significantly change in the presence of either Gag∆p6 or NCp7: 47.5 ± 0.7 bases and 48.4 ± 0.5 bases, respectively (shown in Figure 3B). Thus, hairpin unfolding represents the same two-step opening length in all cases. The opening length is summarized with the rest of the measured data and is shown below in Table 1.

### 3.2. Gag∆p6 Reduces the Energy of Unfolding

During unfolding, the instrument worked to unfold the hairpin and stretch both the dsDNA handles and the newly released ssRNA of the hairpin. Finding the work required to unfold the hairpin (*W_op_*) required subtracting these elasticities. The work of unfolding is the work required to extend the folded (dsDNA only) construct from zero up to the opening force, less the energy required to extend the fully unfolded construct (dsDNA and ssRNA) over the same range (*W_op_* = ∆*G_ds_* − ∆*G_ds+ss_*). There is also a small stiffness correction, as shown in Figure 2B. This calculation is performed for each observed unfolding and folding event and normalized distributions are shown in Figure 3C. However, these distributions are the result of ultrafast force extension/release experiments (Figure 2) and are non-equilibrium; the average work of unfolding does not give the equilibrium unfolding energy of the hairpin (∆*G_op_*). Notably, the equilibrium free energy can be found from these distributions by utilizing several techniques, as discussed in Appendix B. The unfolding energy of TAR RNA is fairly low in these experimental conditions, ∆*G_op_* = 42.1 ± 1.5 k_B_T, reflecting the irregular structure of this hairpin. The addition of Gag∆p6 destabilizes the hairpin further, to ∆*G_op,Gag∆p6_* = 28.9 ± 1.5 k_B_T. This is similar to the effect due to NCp7 seen previously, where ∆*G_op_,_NCp7_* = 28.3 ± 0.9 k_B_T (see Figure 3D for data distributions). Thus, alone, or as a part of Gag, NCp7 further destabilizes an already unstable TAR hairpin. Measured hairpin energies are summarized below in Table 1.

### 3.3. Gag∆p6 Lowers but Does Not Shift the Energy Barrier

Measured distributions of the opening force are plotted in Figure 3E. Fits of these distributions to a kinetic model of hairpin opening produce both the distance to the transition state ($\Delta x_{op}^{†}$) and the barrier height ($\Delta G_{op}^{†}$), as fitted parameters from the solved transition state models described in Appendix C. These fits determine that adding Gag∆p6 lowers the barrier by ~10 k_B_T, an effect similar to that for NCp7 (Figure 3F shows this data). However, Gag∆p6 does not appear to significantly shift the transition state;$\Delta x_{op}^{†}$= 10.9 ± 0.7 nm for TAR alone and $\Delta x_{op,Gag\Delta p6}^{†}$= 9.2 ± 0.7 nm for TAR with Gag∆p6. This contrasts with the shift induced by NCp7, where $\Delta x_{op,NCp7}^{†}$= 4.8 ± 0.6 nm. The corresponding natural (force-free) rate of hairpin unfolding is also returned as a fitted parameter. The slow rate of hairpin opening ($k_{op}^{0}$ = (0.7 ± 0.4) × 10^−8^ s^−1^) is only increased by ~10× for hairpins saturated with Gag∆p6 ($k_{op,\;Gag\Delta p6}^{0}$= (10 ± 6) × 10^−8^ s^−1^). However, NCp7 induced a striking shift in the rate of opening ($k_{op,\;NCp7}^{0}$= (1.2 ± 0.8) × 10^−4^ s^−1^), with a 10^4^x increase in the opening rate. Table 1 summarizes the fitted transition state results.

### 3.4. Landscape Models Locate Gag∆p6 Binding

**Figure 4:**
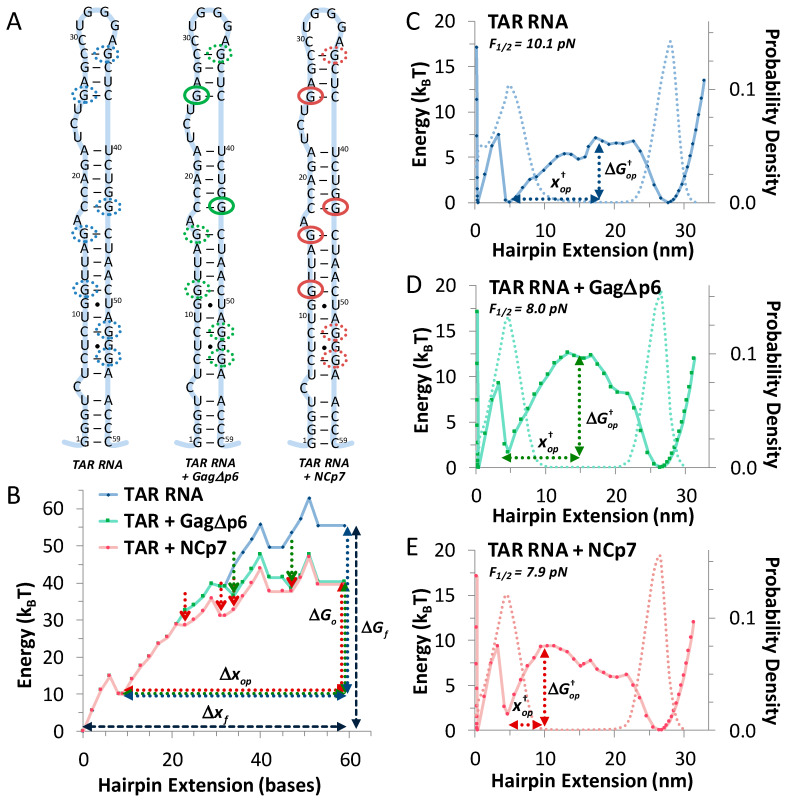
Quantitative modeling of critical protein binding sites. (**A**) Predicted mfold structure for TAR RNA hairpin, highlighting exposed G bases (dotted blue circles). Gag∆p6 binding model incorporates binding to two sites (green–solid circles are confirmed sites in our model), while NC binding is shown for a four-site model (solid red circles). (**B**) Progressive energy of unfolding per extension, as predicted by mfold for TAR (blue), *G_i_*(*N_i_*, *F* = 0). Protein binding is modeled by reducing marked sites (green and red arrows) by a discrete amount (4 k_B_T for NC and 8 k_B_T at each site for Gag∆p6). Predictions for the total energy (∆*G_o_*) and change in length (∆*x_op_*) are shown, compared to the total hairpin energy (∆*G_f_*) and length (∆*x_f_*) from mfold. This unfolding landscape may be ‘tilted’ by the effect of an external force (−*F* ∆*x*). (**C**) Critical force landscapes for TAR (blue), (**D**) TAR + Gag∆p6 (green) and (**E**) TAR + NCp7 (red), including potentials (solid lines for *G_i_*(*N_i_*, *F*_½_), *G*_*i,Gag*∆*p*6_(*N_i_*, *F*_½_) and *G_i,NCp_*_7_(*N_i_*, *F*_½_)) and probability densities (dotted lines), as discussed in the text. The forces, where *P_op_* = *P_cl_* are identified for each, with values for the distance to the opening barrier ($\Delta x_{op}^{†}$) and height ($\Delta G_{op}^{†}$). Predicted state parameters are summarized in Table 1.

Measured landscapes are compared to an mfold-driven model of base pair/stacking stability [41,44,45]. For TAR hairpins (see Figure 4A), the energy associated with each element (canonical base pair, mismatch or loop) *G_i_*(*N_i_*, *F*=0) is determined. Starting from the lower stem (force unfolding begins here), the increasing sum of these energies is found to generally increase with the length of the hairpin as it is gradually destabilized. Figure 4B shows this result for TAR, and the full opening length (∆*x_f_*) requires the full hairpin energy (∆*G_f_*) in the absence of force. During optical tweezers (OT) experiments, the tension applied at the stem increases altering the opening energy along the hairpin length, with –*F* ∆x, effectively ‘tilting’ the landscape to favor the unfolded state. At a critical force (*F_½_*), the energies of the folded and unfolded state are equal, and the probability of seeing the folded and the unfolded state are equal (*P_op_* = *P_cl_, for G_i_*(*N_i_*, *F*_½_)). At *F_½_*, a transition barrier may be quantified and compared to the measured data. This landscape is shown in Figure 4C, and full details of this calculation are found in Appendix D.

Closer examination of the landscape and the location of the unfolded/folded states (determined by the dotted probability density) shows that the lowest part of the stem is destabilized at low force. This ‘fraying’ happens below 4 pN (and is not directly observed in these measurements), and corresponds to the lowest 10–12 bases, which is the difference between the measured length in the experiments (∆*x_op_*) and the natural length of the hairpin (∆*x_f_*). This also corresponds to the difference in the measured hairpin energy (∆*G_op_*) and the mfold predicted value (∆*G_f_*). Experimental data on an artificial DNA hairpin containing a longer stem has confirmed this effect, and there the frayed length was explicitly seen both theoretically and experimentally to unfold at a lower force [23,32]. Here, corrected for fraying, this model matches the data well; explicit comparisons are shown in Table 1 and are discussed below.

Modeling destabilization due to protein binding is somewhat more challenging. We begin with the simplified landscape data measured in OT experiments (the unfolded and the transition state) for TAR in the presence of Gag∆p6. These data are compared to the ‘tilted landscape’ of TAR alone with the goal of altering this landscape to match the experimental data. We begin with the insight that NCp7 binds preferentially to exposed G bases along hairpin irregularities, as shown in previous binding studies [46,47]. Available sites are circled by dotted blue ovals in Figure 4A. Next, the energy difference between the measured unfolding energy for TAR and TAR in the presence of Gag∆p6 is determined (∆*G_op,Gag∆p6_* − ∆*G_op_*). The hairpin energy of destabilization is then applied equally to a combination over *N_b_* binding sites (∆∆*G_op,Gag∆p6_*), so that the total (*N_b_* ∆∆*G_op,Gag∆p6_*) retrieves the destabilization due to Gag∆p6 binding. For each combination, the tilted landscape (*G_i,Gag∆p6_*(*N_i_*, *F*_½_)) gives the position and location of the transition that may be compared to the data (see Appendix D). For Gag∆p6, we find that destabilization is localized to just two binding sites in the upper part of the stem (Figure 4A,B,D), each destabilized by ~8 k_B_T for the total TAR destabilization of 16 k_B_T seen above. This pattern contrasts to the binding of NCp7, which, as we previously showed binds along four sites in the upper stem, completely destabilizing it (Figure 4A,B,E). There, each site was destabilized by ~4 k_B_T for the 16 k_B_T measured difference. All modeled and experimental values are compared in Table 1.

## 4. Discussion

### 4.1. Both NCp7 and Gag∆p6 Chaperone Nucleic Acids

As described in the Methods section, the Gag∆p6 polyprotein studied here is not the full sequence, as it lacks the both the p6 domain on the C-terminus and myristylation at the N-terminus. Thus, the C-terminus of this Gag∆p6-Myr protein (hereafter simply called Gag∆p6) is the NCp9 domain. Here, we compare the effect of this Gag∆p6 construct on TAR hairpin opening to the effects of the completely processed version (NCp7), which we studied previously [23]. RNA interactions with three sequentially processed versions of HIV-1 NC: NCp15, NCp9 and NCp7 have been studied extensively. Both NCp9 and NCp7 lack the anionic unstructured C-terminal p6 domain and are very similar in their ability to bind, aggregate and chaperone the annealing of the complementary structured RNA and DNA molecules, as well as to facilitate reverse transcription [48,49]. In contrast, NCp15 (which contains p6) was shown to have a significantly compromised ability to anneal, aggregate and destabilize nucleic acid (NA) structures, as well as to support reverse transcription [48,49]. We therefore believe that the comparison between the NCp9 domain in the context of Gag and of the completely processed NCp7 protein for their ability to facilitate TAR hairpin unfolding adequately reflects the contribution of the rest of the Gag protein on the nucleic acid chaperone activity of NCp7.

Previous bulk solution studies have shown both NC and Gag strongly facilitate ${\mathrm{tRNA}}_{3}^{\mathrm{Lys}}$ annealing to its primer binding site [50,51,52] and other complementary regions of the structured (-) strand DNA and viral RNA [31,35]. These studies agree that ~100× less Gag is sufficient to maximize nucleic acid annealing compared to NC. This is due to increased binding strength in physiological salt (*K_d_*~100–1000 nM for NC, and *K_d_*~10 nM for Gag) from additional nucleic acid interactions with the MA domain [53,54]. However, the maximal chaperone activity of Gag is still significantly weaker than NC. Furthermore, in contrast to NC, Gag does not facilitate reverse transcription [30,31]. Three major components of the chaperone activity of Gag and NC have been compared: (1) an ability to destabilize nucleic acid (NA) structures, (2) binding kinetics and (3) nucleic acid aggregating activity, which facilitates annealing [11,55]. Gag is just as good as NC in inducing aggregation and also destabilizes nucleic acid structures [52,56]. The inefficiency of Gag compared to NC in facilitating annealing and promoting reverse transcription was associated with slower Gag–NA interaction kinetics, which was attributed to Gag–Gag interactions [30]. However, mutations and deletions in the CA domain of Gag (eliminating most of these interactions) did not allow the chaperone activity of Gag to match NC [31]. Interestingly, it was shown that addition of the polyanion inositol hexakisphosphate (IP6) to Gag–NA complexes significantly improves Gag’s chaperone function, suggesting that IP6 promotes the extended conformation of Gag, with its NC domain bound to NA and its MA domain bound to IP6, similar to the Gag conformation in the immature virion lattice [52,57]. Recent studies show that IP6 can also stabilize the extended multimerized Gag state, facilitating a six-helix bundle formation between the SP1 regions of Gag, located between its CA and NC domains [58]. This contrasts to the compact Gag conformation in which both of its cationic domains, NC and MA, are bound to the same NA molecule. It is possible that either Gag conformation or the binding of its MA domain to RNA limits the chaperone activity of Gag in its compact conformation.

### 4.2. While NCp7 Strongly Facilitates TAR Opening, Gag∆p6 Does Not

**Figure 5:**
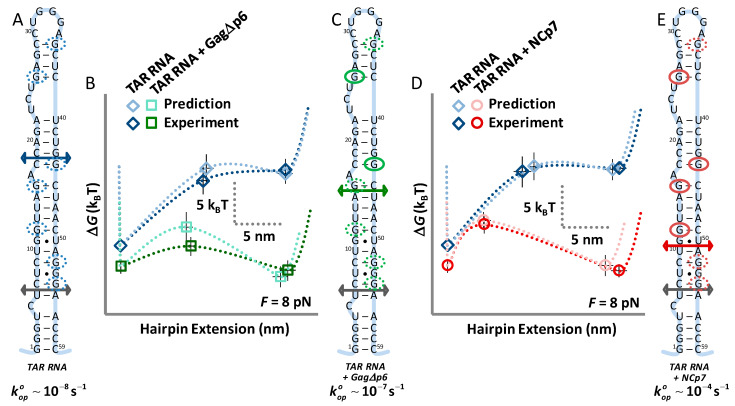
Unlike Gag∆p6, NCp7 dramatically shifts the location of the transition barrier of the TAR hairpin. (**A**) The transition state of this irregular hairpin is marked by an arrow (blue), while exposed G bases are highlighted (blue dotted circles), and the natural rate of hairpin opening is low (10^−8^·s^−1^). (**B**) The measured hairpin landscape (blue) matches values predicted from mfold (cyan), including the effects of hairpin fraying, which unfold the lowest stem (grey arrow). (**C**) Modeled destabilization by Gag∆p6 binding is highlighted (solid green ovals show the sites destabilized by protein binding) and the resulting best match for the unfolding data is compared to TAR in (**B**) (data—green, model—teal). While both the energy of unfolding and the barrier decrease, the transition state is not significantly shifted (green arrow), and the opening rate is only slightly affected (10^−7^·s^−1^). (**D**,**E**) NCp7 destabilizes the entire upper loop (solid red ovals are sites contributing to destabilization), leading to a marked shift in the transition state location (red arrow) toward the folded state (grey arrow) with a corresponding increase in the unfolding rate (10^−4^·s^−1^, data—red, model—pink). Landscapes are shown under the effect of an 8 pN external force. TAR RNA hairpin controls measured in this work and previously are both shown to match mfold predictions.

In this work, we explicitly quantify the destabilizing effect of Gag∆p6 and NCp7 on the TAR hairpin. While the process of complete TAR unzipping from its ends may not occur during (-) DNA strand transfer [8], the details of how Gag∆p6 and NCp7 destabilize this long RNA hairpin with multiple structural defects is expected to contain universal information about the mechanisms by which these chaperone proteins help to restructure nucleic acids. We find that both NCp7 and Gag∆p6 strongly decrease TAR RNA hairpin stability (~16 k_B_T). The differences between Gag∆p6 and NCp7 binding become evident in the transition state data. Structural irregularities in folded TAR RNA, especially those near the apical loop, cause the transition state for unfolding to occur nearly midway down the stem. In contrast, the transition state for a fully matched hairpin is located very near the top of the hairpin [32]. Nonetheless, the natural rate of TAR RNA unfolding is slow, and in the absence of any ancillary proteins, unfolding would be a rare event. The addition of Gag∆p6 does destabilize the hairpin and reduce the force required to unfold TAR. However, only a few sites adjacent to the apical TAR loop are destabilized, leaving the stem end stable, the zero-force transition barrier high, and the opening rate only ~10× faster than in the absence of Gag∆p6 (see Figure 5). In contrast, our previous results on NCp7 showed a uniform destabilization of the upper stem at multiple sites, sharply shifting the transition barrier towards the closed hairpin. Importantly, this decreased the zero-force transition barrier, and increased the natural rate of hairpin opening by 10^4^× (see Figure 5). This finding matched the results of bulk experiments where TAR RNA and DNA annealing increased by 10^4^× in the presence of NCp7 [7].

It appears that NCp7 destabilizes the TAR hairpin by binding to the majority of its available specific binding sites in the loop-adjacent apical part of TAR RNA. Here, we use the prior observation that NC, either as a separate domain or in the context of Gag, has the highest binding specificity for exposed or unstable G bases within NA stem defects. All defects are circled with blue dotted lines in Figure 5A. We can clearly see that NCp7 destabilizes TAR at all its specific sites at the top of the hairpin, thereby increasing the size of the unstable apical loop, which opens cooperatively after stem unzipping to the transition state. As in Figure 5A, in the absence of the protein chaperone, the TAR hairpin must be unzipped through about two thirds of its stem length, up to the C_18_–G_44_ base pair, in order for the rest of the stem to open in a single step. The addition of NCp7 leads to the destabilization of the upper part of the TAR hairpin, so that only about a third of the stem needs to be unzipped prior to the complete opening of the rest of the stem without additional force, moving the transition state to the G_11_–U_50_ base pair (Figure 5E). The net equilibrium unzipping free energy is equally distributed over four specific NC binding sites in the upper part of TAR beyond the transition state, with each site destabilized by ~4 k_B_T. In contrast, the destabilizing effect due to Gag∆p6 appears to be localized only to the most apical loop-adjacent specific sites, as shown in Figure 5C. TAR still must be unzipped through about half of its stem length for the rest of the hairpin to open cooperatively, such that the transition state for unzipping now resides around the A_15_–U_46_ base pair.

Why would the effect of Gag∆p6 on TAR stability be spatially limited, compared to NCp7, despite its lower binding specificity? There are two mutually non-exclusive possible explanations for this observation. One is that each Gag∆p6 molecule is bound to TAR in its compact state with its NC and MA domains, thereby occupying a much larger binding site of approximately 20–30 nucleotides [52]. It is possible that the 59-nucleotide-long TAR RNA can only accommodate two Gag molecules, leading to the binding of only two NC domains, thereby destabilizing only the two highest affinity sites at the top of the TAR stem. The MA domain of Gag does not participate in gRNA selection for packaging through direct preferential binding and does not have chaperone activity on its own [49,59]. Therefore, only the specific binding of the NC domain of Gag, but not of MA, can lead to hairpin destabilization. At the same time, NCp7 is known to bind every ~7 nucleotides of any nucleic acid at saturation, such that multiple NCp7 molecules, up to a total of ~8–10 molecules, can bind TAR RNA. Not all these NCp7 molecules will bind specific sites and destabilize them, and the destabilization will be limited to the highest affinity locations, which, in the case of TAR, appears to be the four loop-adjacent sites. Another possible reason for the site-limited effect of Gag∆p6 binding could be the higher mobility of NCp7 when bound to RNA. While detailed kinetics of NCp7 and Gag∆p6 binding are unknown, it is clear that NC is highly mobile in its bound state [11]. This means that NCp7 can likely be “delocalized” over several specific sites, thereby destabilizing each of them during the timescale of our unzipping experiments (over ~2 s), while the less mobile Gag∆p6 remains permanently bound on the same timescale and destabilized in only the most specific TAR locations.

Our data can be placed in the context of a recent study of Gag binding to the 5′UTR [60,61], which shows that the majority of sites within the Psi RNA region that crosslink to Gag also show significant concurrent changes in their RNA secondary structure. How is it possible that the same Gag protein binding leads to two opposite effects on the local RNA structure? The most plausible answer is that, while specific interactions between the NC domain of Gag with destabilized G residues lead to local structure destabilization, the binding of the MA domain most likely leads to a local increase in duplex stability, as it does for most non-specific multivalent cation binding interactions. Thus, the combined effect of general local destabilization and the stabilization of particular RNA bases at sites of Gag crosslinking most likely reflects the pattern of NC and MA domain binding. Previous studies of the same region of gRNA, performed on ex virio gRNA derived from infected cells in which the NC fingers in Gag were exposed to chelating agents, also shows that wild type Gag binding to RNA can either destabilize or stabilize a local RNA structure [62].

In summary, the HIV-1 NC protein, in the context of Gag, is still able to bind RNA with modest selectivity and to locally destabilize RNA structure at a few specific sites (even more strongly than NC at these sites), while leaving the rest of the RNA unperturbed, or even slightly more stable, thereby leaving high free energy barriers to NA re-folding, and leading to much longer times for NA rearrangement to achieve its most stable conformation. This contrasts with the processed NCp7 protein, which binds and slightly destabilizes the majority of its weakly specific sites, most likely by moving between these sites on a relatively fast time scale and leading to the significant facilitation of the NA restructuring rate. While the first activity of NC in the context of Gag is optimal for selective gRNA packaging, the second activity of the processed NCp7 is optimized for facilitating the refolding of any nucleic acid into its optimal lowest free energy conformation. Therefore, these results demonstrate how NCp7, only after cleavage from Gag, is optimized for nucleic acid chaperone activity during reverse transcription.

## Figures and Tables

**Table 1:** Summary of experimental OT results and modeled mfold data for TAR unfolding and TAR in the presence of Gag∆p6. Data is compared to previous results for TAR in the presence of NCp7. The opening length (∆*x_op_*) and energy (∆*G_op_*) are calculated from OT and mfold data and are independent of force. The distance to the barrier ($\Delta x_{op}^{†}$) and the height ($\Delta G_{op}^{†}$) are measured in OT experiments and calculated in mfold at the force *F_½_*, where the probability of observing unfolding/folding is equal (*P_op_* = *P_cl_*). The natural rate of opening ($k_{op}^{0}$) in the absence of force was also found from OT data and reflects the instability of the TAR hairpin in the presence of NCp7 relative to the TAR and TAR + Gag∆p6.

Experiment	Δ*x_op_*	Δ*G_op_*	*F*_½_	$\Delta x_{op}^{†}$	$\Delta G_{op}^{†}$	$k_{op}^{0}$
	(bases)	(k_B_T)	(pN)	(nm)	(k_B_T)	(s^−1^)
TAR (OT)	48.2 ± 0.6	42.1 ± 1.5	10.6 ± 0.2	10.9 ± 0.7	28.0 ± 1.4	(0.7 ± 0.4) × 10^−8^
TAR (mfold)	48 ± 1	41.5 ± 1.5	10.1 ± 0.1	11.3 ± 0.9	30.7 ± 2.0	-
TAR + Gag∆p6 (OT)	47.5 ± 0.7	28.9 ± 1.5	7.7 ± 0.2	9.2 ± 0.7	18.1 ± 0.9	(10 ± 6) × 10^−8^
TAR + Gag∆p6 (mfold)	48 ± 1	26.0 ± 1.5	8.0 ± 0.2	8.6 ± 1.0	20.1 ± 1.5	
TAR + NCp7 (OT)	48.4 ± 0.5	28.3 ± 0.9	7.7 ± 0.2	4.8 ± 0.6	14.3 ± 1.3	(1.2 ± 0.8) × 10^−4^
TAR + NCp7 (mfold)	48 ± 1	25.6 ± 1.5	7.9 ± 0.1	4.7 ± 0.9	14.8 ± 1.2	-

Results were determined from data shown in Figure 3 for TAR RNA and TAR + Gag∆p6, while TAR + NCp7 was found in a previous work [23] and are shown here for comparison. In the previous work, the parameters measured for the TAR RNA hairpin were also found and were all the same as the parameters found in this study within measured uncertainty (those results are not shown). All errors are the standard error of the mean (SEM).

## Appendix A. Polymer Models of Elasticity

The elastic response of our long dsDNA handles under tension are modeled with the extensible Worm-Like Chain, which was solved in the high-force limit [63,64,65]:

(A1) $$b_{ds}(F)=B_{ds}\left[1-\frac{1}{2}{\left(\frac{k_{B}T}{P_{ds}F}\right)}^{1/2}+\frac{F}{S_{ds}}\right].$$

The measured extension under force (*b_ds_*(*F*)) may be modeled to the data through fitted parameters that have been found for dsDNA over many experimental conditions, including changing salt, pH and known variations due to DNA length and trap stiffness [66,67,68,69]. The handles used in these studies are similar to those used previously and are well described by the fitted polymer parameters: *B_ds_* = 0.340 ± 0.001 nm/bp, *P_ds_* = 40 ± 3 nm and *S_ds_* = 800 ± 200 pN) [23,32]. These values were fixed throughout the analysis, and effectively describe the extension data of the two dsDNA handles at forces below the opening transition (Figure 2B).

The Freely Jointed Chain Model is appropriate for polymers with greater rotational freedom at each element, and is commonly used to characterize single stranded nucleic acids [65]:

(A2) $$b_{ss}(F)=B_{ss}\left[\coth{\left(\frac{2P_{ss}F}{k_{B}T}\right)}^{1/2}-\frac{1}{2}\frac{k_{B}T}{P_{ss}F}\right]\cdot \left[1+\frac{F}{S_{ss}}\right].$$

Though there is a small range of values that adequately describe RNA, the values used here, *B_ss_* = 0.590 ± 0.001 nm/bp, *P_ss_* = 1.4 ± 0.3 nm and *S_ss_* = 800 ± 100 pN have been used before and give consistent results [70]. This model describes the 59-base TAR RNA. A linear combination of this with the Worm-Like Chain (WLC) above describes the extension curves after unfolding (*b_ds_*(*F*) + *b_ss_*(*F*), Figure 2B). In practice, the total change in extension upon unfolding (∆*x_op_*) is easily discerned by finding *b_ds_*(*F_op_*) + *b_ss_*(*F_op_*) and subtracting *b_ds_*(*F_op_*). From this force-dependent length, the number of bases (independent of force) may be recovered (Figure 3A). This approach may also apply to cycles of extension and release where the elasticity of the handles is difficult to model due to nonspecific interactions with the beads.

Comparisons with hairpins saturated with NCp7 are taken from a previous work, though that work covered several loading rates. To simplify the comparison, only data taken at the same loading rate as this work (10 pN/s) was shown from the full set. Furthermore, the data for the native TAR RNA hairpins found here matches the results from previous studies within parameters of uncertainty [23,70].

## Appendix B. Equilibrium Energies from Non-Equilibrium Measurements

As shown in the Methods section, the work performed by the instrument during unfolding (*W_op_*) and folding (*W_cl_*) is determined for each cycle. Though these measurements exclude contributions due to polymer elasticity, they are not equilibrium measurements of the hairpin energy. To recover the equilibrium value, probability density distributions were collected for work measured during folding *P_cl_*(*W*) and unfolding *P_op_*(*W*). Importantly, the integral of these distributions over the *x*-axis is unity. If these experiments were in equilibrium, the peak of these distributions would represent the free energy of the hairpin. Instead, these non-equilibrium distributions cross at precisely the equilibrium energy of hairpin unfolding ∆*G_o_* [71,72]:

(A3) $$\frac{P_{op}(W)}{P_{cl}(W)}=e^{\left(W-\Delta G_{o}\right)/k_{B}T}.$$

These crossing points are labeled in Figure 3B along the energy axis. Though other techniques may be used to deduce the equilibrium energy, they do not appear to deliver more precise results for the data shown here [73,74]. Due to handle–bead interactions and Gag’s affinity for single-stranded RNA, closing events were not always seen at the loading rates shown here. However, the hairpin energy of TAR matches that seen previously (within uncertainty) and the energy in the presence of Gag∆p6 appears close to the value seen in NCp7 (the RNA binding domain of Gag∆p6) [23,70].

## Appendix C. Kinetic Models and the Transition State

The distributions of the measured opening forces will give information about the distance to the barrier ($\Delta x_{op}^{†}$) and the height ($\Delta G_{op}^{†}$) of the barrier to unfolding [75,76,77]. For a simple two-state model of hairpin unfolding (as seen in this work), exact solutions to the reaction rate (in this case, the rate of hairpin opening) can be solved, even under the influence of external force (*k_op_*(*F*)), which increases at a known rate (*r*, which is fixed at 10 pN/s in these experiments),

(A4) kop(F)=kopo(1−xop†FΔGop†ν)1ν−1exp{ΔGop†kBT[1−(1−xop†FΔGop†ν)1ν]},

and the corresponding probability of observing hairpin opening at any force (*P_op_*(*F*)) is then,

(A5) Pop(F)=kop(F)rexp{kopoxop†r−kop(F)xop†r(1−xop†FΔGop†ν)1−1ν}.

The zero force (natural) rate of hairpin opening ($k_{op}^{0}$) is another independent fitting parameter, though, in practice, limited combinations of the rate, the barrier height, and the distance are physically possible. Both the rate and the probability bear explicit solutions only for two specific potential surfaces (*ν* = 1/2 for a cubic potential and *ν* = 2/3 for a cusp shaped potential). Furthermore, these equations are difficult to fit and non-physical local minima may be seen during minimization over a robust parameter space. To speed up the fitting process, simplified versions of these expressions relate the peak of the force distribution ($P_{op}^{\max}$, with units of pN^−1^, and located at the force $F_{op}^{\max}$ on Figure 3E);

(A6) $$\frac{\Delta G_{op}^{†}}{k_{B}T}=\frac{F_{op}^{\max}x_{op}^{†}}{k_{B}T}\left[\frac{x_{op}^{†}}{x_{op}^{†}-P_{op}^{\max}k_{B}T\cdot e}\left(1-\nu \right)\right].$$

This expression gives reasonable starting values for the barrier height ($\Delta G_{op}^{†}$) and distance ($\Delta x_{op}^{†}$), in the fits for the final values. Fits are shown in Figure 3C and fitted parameters are found in Table 1, while all fits yield typical values of $\chi_{\upsilon }^{2}∼1$. Typically, fits to the different shape factors give different fitted results, and it is customary to fit to both factors and average the results. The averages are shown in Table 1, with propagated uncertainty. Averaged parameters for TAR RNA matched previous results within uncertainty [23]. For the shape factor *ν* = 2/3, fits to the TAR + Gag∆p6 distributions returned parameters with higher uncertainty, as there were many local minima with similar values of $\chi_{\upsilon }^{2}$. This may be due to Gag∆p6 binding to handles and to the unfolded hairpin. Thus, a weighted average was used to combine the data from the two shape factors. Distributions of the hairpin closing forces are not characterized here, as Gag∆p6 binding to ssRNA complicates a multi-step re-folding pathway [78].

## Appendix D. Modeling the Energy Landscape

As discussed in the text, mfold-based models derive the predicted values of the barrier location and height to compare to fitted data of the unfolding forces. Figure 4B shows the summed energies *G_i_*(*N_i_*, *F*=0), and Figure 4C shows them for the TAR hairpin under the influence of force, and *G_i_*(*N_i_*, *F*_½_) is where the probability of finding the hairpin open or closed is equal. The landscapes are modified to include a Morse potential $∼\;D\cdot {\left(1-e^{-ax}\right)}^{2}$ and the elasticity of ssRNA $∼\;k\cdot x^{2}$. This modified landscape is then used to calculate the probability densities of each state ($p∼e^{-G_{i}\left(F\right)}$, and this is integrated over the state) [44,45,79]. This critical force landscape also highlights the lower stem fraying, as discussed in the text.

Various combinations of G-base destabilizations give changed landscapes (*G_i,Gag∆p6_*(*N_i_*, *F*_½_) and *G_i,NCp7_*(*N_i_*, *F*_½_)) and different combinations of the barrier location and height ($\Delta x_{op}^{†}$ and $\Delta G_{op}^{†}$) that are compared to the data. The location of destabilizing sites may be quickly deduced from the single molecule data for the hairpin compared to the hairpin under the influence of the ligand. Generally, ligand binding distributed evenly along the hairpin chiefly alters the energy of unfolding (this work cannot resolve different affinities between different sites). Ligand binding localized near the lower stem induces further fraying and reduces all the measured parameters, including the observed unfolding length. However, if binding is concentrated near the apical loop, the unfolding length is not affected, while the distance to the barrier is reduced, as only the lowest part of the stem needs to be destabilized [79]. The data for TAR RNA in the presence of Gag∆p6 and NCp7 reveals changes in the transition state (and unfolding energy), but no change in the unfolding length, with the difference lying in the negligible amount of transition state shift. Therefore, binding, which is already restricted to exposed G-bases, must occur at those bases near the loop. The best fits for both cases are shown in Figure 5. In those cases, multiple combinations of sites were tried, but those shown in Figure 5 best matched the data for the transition state [23]. A four-site model with ~4 k_B_T destabilization at each site (to match the total measured ~16 k_B_T for NCp7 binding) best fits the data for NC. This binding model destabilizes the upper loop and reproduces the transition state shift. However, only a two-site model works best for Gag∆p6, with ~8 k_B_T destabilization at each site (to, again, match the total measured ~16 k_B_T energy difference due to Gag∆p6 binding). This model reduces the transition state height, but only modestly changes the transition state location.
